# Upcycling Municipal Solid Incineration Fly Ash into Layered Double Hydroxide Nanomaterials: Heavy Metal Immobilization and Environmental Risk Assessment

**DOI:** 10.3390/nano16110697

**Published:** 2026-06-03

**Authors:** Yue Zhao, Xiaona Wang, Ze Zhang, Menglan Xu

**Affiliations:** 1 State Key Laboratory of Water Resource Protection and Utilization in Coal Mining, China Energy Investment Group, Beijing 102209, China; 15865952895@163.com; 2 School of Energy and Environmental Engineering, University of Science and Technology Beijing, Beijing 100083, China; wangxiaona071@163.com; 3 Shunde Innovation School, University of Science and Technology Beijing, Foshan 528399, China; 4 Graduate School of Frontier Sciences, The University of Tokyo, Kashiwa 277-8563, Japan; 4376795547@edu.k.u-tokyo.ac.jp; 5 State Key Laboratory of Iron and Steel Industry Environmental Protection, Central Research Institute of Building and Construction Co., Ltd., MCC Group, Beijing 100088, China

**Keywords:** MSWI FA, layered double hydroxides, heavy metal stabilization, sodium dimethyl dithiocarbamate, environmental risk assessment, FA waste utilization

## Abstract

Municipal solid waste incineration fly ash (MSWI FA) represents a significant environmental challenge due to its high content of toxic heavy metal (HM) and large-scale generation. This study demonstrates the feasibility pathway for converting hazardous MSWI FA into well-crystallized layered double hydroxide nanosheets (LDH-FA). Sodium dimethyl dithiocarbamate (SDD) was incorporated as a chelating stabilizer to enable synergistic HM immobilization during acid leaching and crystallization. High-resolution transmission electron microscopy (HRTEM) confirmed the characteristic two-dimensional nanosheet morphology with interlayer spacings consistent with LDH structures, while elemental mapping revealed homogeneous distribution of Pb and Zn within the nanosheet matrix. SDD dosages higher than 1.0 wt% effectively suppressed HM leaching, and Pb concentrations were controlled below 0.1 mg/L and Zn maintained at minimal levels. BCR sequential extraction analysis further demonstrated that SDD treatment effectively transformed HMs from bioavailable acid-soluble fractions to stable forms. This investigation establishes an innovative approach to MSWI FA resource utilization and provides mechanistic insights into HM stabilization within LDH nanostructures, offering a scientific basis for safer applications of waste-derived nanomaterials.

## 1. Introduction

Rapid urbanization and economic development have led to a dramatic increase in municipal solid waste generation, which has reached 2.01 billion tons annually and is projected to increase to 3.5 billion tons by 2050 [1]. Municipal solid waste incineration (MSWI) has emerged as an effective waste treatment technology, offering significant volume reduction up to 90% and mass reduction of 70–75% [2]. However, this process generates substantial amounts of MSWI fly ash (hereafter referred to as FA) as a hazardous by-product, accounting for 3–5% of the initial waste mass [3]. Owing to its fine particle size and enriched toxic constituents, FA poses non-negligible environmental and human-health risks [4]. Accordingly, the safe management and sustainable valorization of FA remain a critical challenge for modern waste management systems. FA exhibits complex physicochemical properties with a heterogeneous composition. The major constituents include SiO_2_ (15–30%), Al_2_O_3_ (5–15%), Fe_2_O_3_ (2–10%), and CaO (20–35%) [5,6]. This unique mineral composition endows FA with potential mechanical strength and catalytic properties, making it theoretically suitable for various applications such as construction materials and environmental remediation [7]. However, FA is classified as hazardous waste in numerous jurisdictions; the primary environmental risks stem from the presence of leachable heavy metals (HMs) such as Pb, Zn, Cu, Cr, and Cd [8]. These metals can readily mobilize under various environmental conditions, potentially contaminating soil and groundwater systems.

Current management strategies for FA include cement-based solidification/stabilization prior to landfilling, incorporation into geopolymers and construction materials, and thermal treatments such as vitrification [7,8]. While these approaches partially mitigate the FA disposal burden, they generally fail to fully stabilize the intrinsic HMs under long-term environmental exposure or do not yield high-value functional products [5]. This limitation underscores the need for an integrated strategy that concurrently achieves complete HM immobilization and converts FA into value-added materials. From a resource-recovery perspective, the abundance of metal-bearing components (e.g., Ca, and Fe) provides a favorable chemical basis for synthesizing layered double hydroxides (LDHs) [9,10]. LDHs are two-dimensional nanostructured anionic clays composed of positively charged brucite-like layers and charge-compensating interlayer anions, widely explored as high-performance adsorbents and reactive media for pollutant control [11,12]. The unique structural features make LDHs particularly appealing for designing multifunctional sorbents derived from complex solid waste [13,14]. A central barrier, however, is that intrinsic HMs in FA may be redistributed or activated during alkaline dissolution–reprecipitation, potentially increasing leachability and compromising environmental safety [15,16,17]. Chelating agents (CAs) such as sodium dimethyl dithiocarbamate (SDD) can reduce metal mobility by forming stable coordination complexes [18,19,20]. SDD contains dithiocarbamate functional groups (–NC(S)S^−^) that act as bidentate ligands, coordinating with divalent heavy metal ions through both sulfur donor atoms to form highly stable, water-insoluble chelate complexes, thereby effectively suppressing metal leaching under environmental conditions [18,19,20]. We hypothesize that integrating chelation with LDH-directed synthesis yields synergistic immobilization: chelation constrains metal speciation at the molecular scale, while the LDH nanostructure provides complementary sequestration via surface binding, interlayer confinement, and incorporation into hydroxide/secondary mineral phases [21,22], simultaneously mitigating environmental risk and enabling value-added FA upcycling.

To realize this concept, FA was employed as a precursor for the synthesis of nanostructured CaFe-LDH materials (denoted as LDH-FA), with sodium dimethyl dithiocarbamate (SDD) introduced in situ during hydrothermal synthesis to enable HM stabilization and LDH crystallization. The study systematically (i) optimized key synthesis parameters governing LDH-FA nanocrystal formation; (ii) characterized the nanoscale morphology, phase composition, and surface/interlayer chemical features of the resulting materials; (iii) evaluated HM leaching behavior and quantified the associated environmental risk by integrating leaching tests with BCR sequential extraction and risk assessment code (RAC) methodologies. This integrated investigation not only demonstrates a safer upcycling route for hazardous FA into environmentally compatible LDH-based nanomaterials, but also explicitly bridges materials chemistry with environmental risk science, thereby contributing to the risk-informed design of waste-derived functional materials for environmental remediation.

## 2. Materials and Methods

### 2.1. Materials

The FA used in this study was obtained from a waste incineration plant in Beijing, China. All chemical reagents, including sodium hydroxide (NaOH), hydrochloric acid (HCl), iron chloride (FeCl_3_), SDD, acetic acid (CH_3_COOH), sulfuric acid (H_2_SO_4_), and nitric acid (HNO_3_), were of analytical grade and purchased from Sinopharm Chemical Reagent Co., Ltd., Shanghai, China. All aqueous solutions were prepared using deionized water produced by a Milli-Q system (18.2 MΩ·cm, Millipore, Burlington, MA, USA).

### 2.2. Synthesis of LDH from FA (LDH-FA)

LDHs were synthesized through acid-leaching followed by a precipitation method. Initially, FA was immersed in 3 M HCl for 2 h to dissolve amorphous silica. The resulting solution was filtered through a 0.45 μm cellulose acetate membrane filter to remove silicic acid colloids. SDD was added to the filtrate at different weight ratios (0, 0.5, 1, and 2 wt%), followed by mixing with FeCl_3_ at various weight ratios (FA:FeCl_3_ = 1:1, 2:1, and 3:1). NaOH solution was gradually added to adjust the pH to 11.5. The mixture was then hydrothermally treated in an autoclave at 70 °C for 12 h. After treatment, the product was centrifuged at 6000 rpm for 10 min and washed three times with Milli-Q water to remove excess NaOH, yielding LDH materials with different compositional ratios.

### 2.3. Characterization

The obtained samples were characterized by X-ray diffraction (XRD), Raman spectroscopy, and Fourier transform infrared spectroscopy (FTIR). XRD analysis was performed using a Rigaku D/max2500 X-ray diffractometer with Cu Kα radiation (λ = 1.540 56 Å, Rigaku, Japan). FTIR measurements were conducted on a Nicolet 380 FTIR spectrometer (Thermo Electron Corporation, Waltham, MA, USA). Raman spectroscopic analysis was carried out using a Thermo DXR microscope confocal laser micro-Raman spectrometer (Thermo Fisher Scientific, USA) equipped with a 532 nm laser. The bulk elemental composition of the raw FA was determined by X-ray fluorescence (XRF) spectrometry (Malvern Panalytical, Almelo, The Netherlands).

### 2.4. Leaching Toxicity Experiments and Heavy Metal Speciation Analysis

Using deionized water and mixed sulfuric–nitric acid solution (simulating acid rain) as leaching agents, the concentrations of HMs leached from LDH-FA in both extractants (liquid–solid ratio of 10:1) were compared with the Class IV limits specified in the Standard for Groundwater Quality (GB/T 14848-2017) [23] to evaluate the environmental compatibility and long-term safety of LDH-FA in practical applications (e.g., in situ groundwater remediation). The obtained liquid samples were analyzed for HMs using ICP-AES [24].

The chemical speciation of HMs in LDH-FA was analyzed using the four-step sequential extraction procedure proposed by the European Community Bureau of Reference (BCR) (Table S1). This method categorizes HMs into four fractions: acid-soluble fraction (F1), reducible fraction (F2), oxidizable fraction (F3), and residual fraction (F4) [25].

The risk assessment code (RAC) independently determines the potential environmental risk level of various HMs [26,27]. Based on the readily bioavailable portion of the solid-phase matrix HMs fractions, which is calculated as shown in Equation (1), the classification of the environmental risk level of HMs is shown in Table S2.

(1) $$\mathrm{RAC}=\frac{F1}{F1+F2+F3+F4}\;\text{×}\;100$$

where F1, F2, F3 and F4 represent the acid-soluble, oxidizable, reducible and residual fractions, respectively, mg/kg.

## 3. Results and Discussion

### 3.1. Compositional and Structural Characterization

The oxide composition analysis (Table 1) revealed that calcium oxide (CaO) constitutes the primary oxide component of raw FA, with additional components including chloride salts (such as KCl and NaCl), sulfates, sodium oxide (Na_2_O), potassium oxide (K_2_O), silicon dioxide (SiO_2_), and minor quantities of other metal oxides. X-ray diffraction (XRD) analysis (Figure S1) demonstrated that the diffraction pattern of raw FA exhibited characteristic peaks of multiple crystalline phases, including Ca(OH)_2_, KCl, NaCl, CaSO_4_·2H_2_O, CaClOH, and CaCO_3_, which collectively confirm that calcium-based compounds, alkali metal salts, and silicates represent the predominant constituents in raw FA.

As shown in Table S3, Zn was the most abundant HM, with a concentration of 3936 mg/kg, followed by Pb, and other HMs. Therefore, Zn and Pb were selected as representative target metals because Zn was the dominant HM in the raw FA, while Pb is of environmental concern due to its high toxicity.

Following acid leaching and hydrothermal treatment, the XRD patterns of synthesized LDH materials with different FA-to-FeCl_3_ ratios exhibited significant structural transformations (Figure S2). The characteristic diffraction peaks changed markedly, and new reflections appeared at 2θ value of 11.3, 22.8, 23.2, 38.4 and 54.1°, which are consistent with the typical pattern of CaFe-LDH, confirming that CaFe-LDH was successfully synthesized [28]. As the FA-to-FeCl_3_ ratio increased from 1:1 to 3:1, the intensity of the LDH characteristic peaks slightly enhanced, although the fundamental structure remained similar.

**Figure 1:**
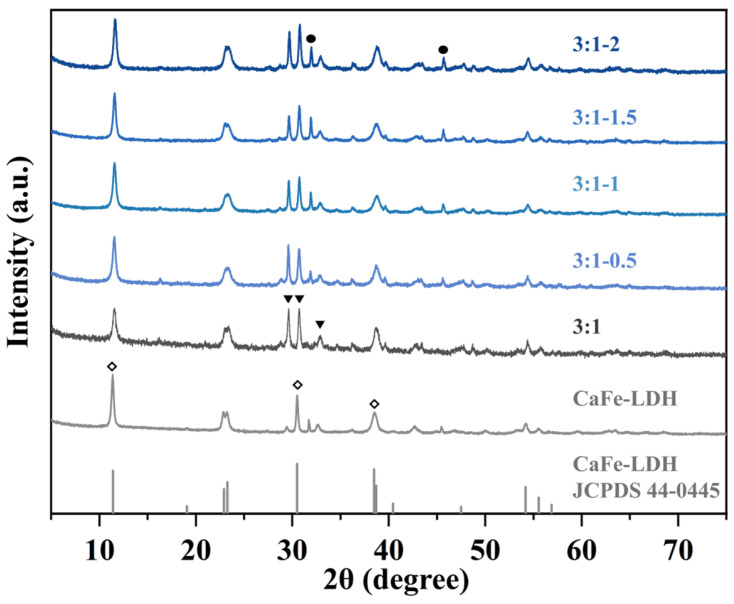
XRD patterns of LDH materials with different SDD dosages.

Figure 1 shows the XRD patterns of CaFe-LDH synthesized from CaCl_2_·6H_2_O and FeCl_3_ (Ca:Fe = 3:1) and those derived from FA with the same ratio under different SDD dosages (0.5–2.0 wt%). The CaFe-LDH pattern matches well with the standard card (JCPDS 44-0445) [29], displaying typical LDH reflections at 2θ value of 11.3, 22.8, 23.2, 38.4 and 54.1°. FA-derived samples exhibit similar LDH diffraction peaks, confirming that FA can effectively act as a Ca source for LDH formation. The slightly broadened profiles and additional weak signals near 28.7, 29.6, 30.8, and 32.9° suggest the presence of minor silicate and ferrite phases inherited from the ash matrix, indicating partial incorporation of FA minerals into the LDH-containing system. With SDD addition, the LDH peaks become progressively sharper and more intense. Quantitative analysis of the (003) reflection confirms a reduction in its full width at half maximum, indicating enhanced crystallinity and growth of more coherent nanoscale layered domains along the stacking direction. The improved long-range structural order likely provides a more stable host matrix for HMs, whether incorporated within the hydroxide layers or confined as SDD–metal complexes in the interlayer region, which is consistent with the significantly reduced Pb leaching observed at SDD dosages ≥ 1.0 wt%. At higher SDD levels, faint new reflections appear at around 31.8°and 45.7°, attributable to minor secondary phases formed through metal–SDD coordination.

**Figure 2:**
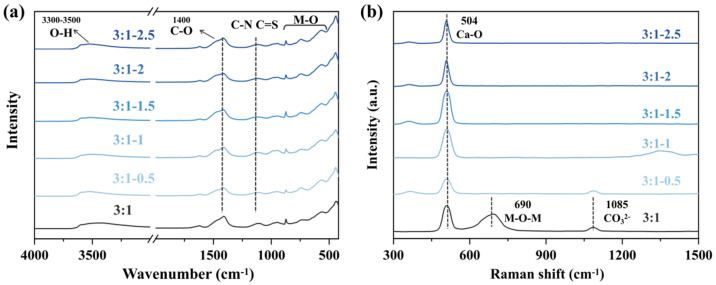
FTIR analysis (**a**) and Raman spectra (**b**) of LDH materials with various SDD dosages.

FTIR spectra (Figure S3) revealed clear variations in absorption features between raw FA and the synthesized LDH-FA materials with different ratios (FA:FeCl_3_ = 1:1, 2:1, and 3:1). All samples exhibited a broad band at 3400–3500 cm^−1^, attributed to O-H stretching vibrations [30]. A weak band at approximately 1630 cm^−1^ is assigned to the H-O-H bending vibration of interlayer water molecules, further confirming the hydrated nature of the LDH interlayer gallery. This band was more intense in LDH specimens, suggesting increased interlayer water and hydroxyl content during synthesis. A distinct peak near 1400 cm^−1^ corresponds to the asymmetric stretching of interlayer CO_3_^2−^ anions, characteristic of LDH structures [30]. With higher FA/FeCl_3_ ratios from 1:1 to 3:1, subtle enhancement of the bands in the 500–700 cm^−1^ region was observed, reflecting strengthened metal–oxygen (M-O) interactions and slight rearrangement within the metal hydroxide layers. FTIR features confirm the successful conversion of FA into LDH materials possessing well-defined layered frameworks. Figure 2a shows the FTIR spectra of LDH-FA materials (FA:FeCl_3_ = 3:1) and varying SDD dosages (0.5–2.5 wt%). A broad band at 3500–3300 cm^−1^ corresponds to O-H stretching of hydroxyl groups and interlayer water, with slight shape variations at higher SDD levels suggesting changes in interlayer hydration. The strong peak near 1400 cm^−1^ arises from asymmetric CO_3_^2−^ stretching, and the interlayer anion, whose minor intensity and position shifts indicate anionic rearrangement, possibly due to partial exchange with SDD-derived species. At 1000–500 cm^−1^, characteristic M-O and M-OH vibrations appear with negligible variation, implying limited structural alteration but subtle changes in local metal coordination. Weak new bands at 1200–1100 cm^−1^ are assigned to C-N and C=S stretching from SDD, confirming its incorporation [31]. The slight shift of these bands to lower wavenumbers relative to free SDD indicates that the thiocarbamate sulfur atoms coordinate with metal ions, forming stable chelate complexes within the LDH matrix. Collectively, these results indicate that SDD tunes the interlayer environment, slightly modifies metal–oxygen bonding, and introduces functional groups capable of forming stable metal complexes, consistent with enhanced crystallinity and HM immobilization observed in XRD patterns.

Raman spectroscopy was employed to characterize raw FA and LDH-FA materials with different ratios (FA:FeCl_3_ = 1:1, 2:1, and 3:1) within 150–1500 cm^−1^ (Figure S4). Raw FA shows pronounced peaks at 550–600 cm^−1^ and 1080–1100 cm^−1^, assigned to Ca-O and symmetric CO_3_^2−^ stretching vibrations, confirming calcium-based compounds [32]. After LDH formation, the spectra change markedly, with diminished carbonate peaks and new bands at 700–800 cm^−1^ attributable to M-O-M vibrations [32], evidencing layered structure development. Increasing the FA to FeCl_3_ ratio from 1:1 to 3:1 causes slight shifts and intensity changes of these features, reflecting minor structural variations among LDHs. Overall, Raman results corroborate the XRD and FTIR analyses, confirming the transformation of FA into LDH with well-defined layered structures. The LDH prepared at 3:1 ratio display more ordered and stable features, favorable for improved performance and reduced HM leaching in environmental applications. Figure 2b compares the Raman spectra of LDH-FA materials with various SDD dosages. The unmodified sample shows distinct bands at 504 cm^−1^, 690 cm^−1^, and 1085 cm^−1^, attributed to Ca-O, M-O-M lattice vibrations, and the asymmetric stretching of interlayer CO_3_^2−^, respectively. With increasing SDD dosage, the Ca-O band remains nearly constant, whereas the 690 cm^−1^ feature progressively diminishes and finally vanishes, indicating the breakdown of metal–oxygen-metal linkages. This disappearance signifies that certain metal oxides from FA engage in coordination with SDD, forming stable metal–SDD complexes embedded within the LDH matrix. The slight attenuation of the CO_3_^2−^ band at 1085 cm^−1^ further reflects the partial exchange or rearrangement of interlayer anions. Collectively, these spectral evolutions suggest that SDD alters the local structure of LDHs by consuming reactive metal–oxygen species and promoting chelation, thus stabilizing metal ions and reducing their leachability. The BET specific surface areas and pore structure parameters of the LDH-FA materials are provided in Table S3. The surface areas ranged from 17.5 to 20.3 m^2^/g and did not vary systematically with SDD dosage, indicating that SDD incorporation primarily modifies the chemical coordination environment rather than the physical textural properties of the LDH nanosheets.

**Figure 3:**
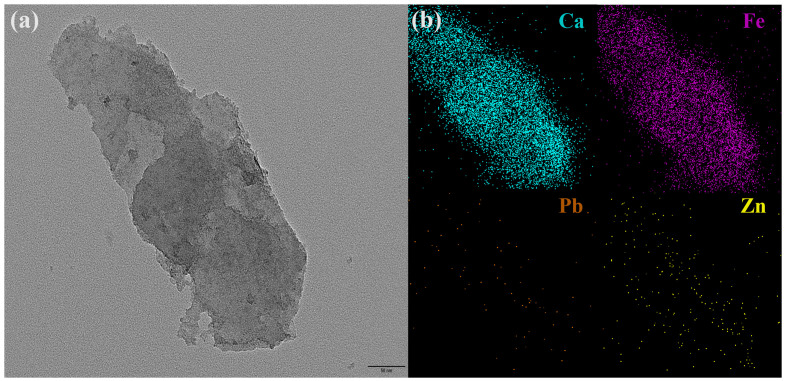
TEM spectra (**a**) and element distribution spectra (**b**) of LDH-FA Materials with 2.5 wt% SDD dosages.

TEM characterization revealed the microstructural and elemental distribution characteristics of the LDH-FA (FA:FeCl_3_ = 3:1; SDD = 2.5 wt%). The HRTEM image (Figure 3a) reveals a well-defined lamellar morphology with characteristic nanoscale sheet-like layers, consistent with the two-dimensional nanostructure of LDH. The element mapping (Figure 3b) shows that Pb and Zn are homogeneously distributed throughout the LDH-FA nanosheets [33], coexisting with Ca and Fe. Notably, this uniform nanoscale dispersion has raised significant concerns regarding the potential leaching risk of HMs. While LDH materials are conventionally considered to possess excellent HM immobilization capabilities, the nanoscale integration of Pb and Zn within the layered structure may potentially enhance their environmental mobility and release risk [34,35].

### 3.2. Heavy Metal Immobilization and Leaching Behavior

**Figure 4:**
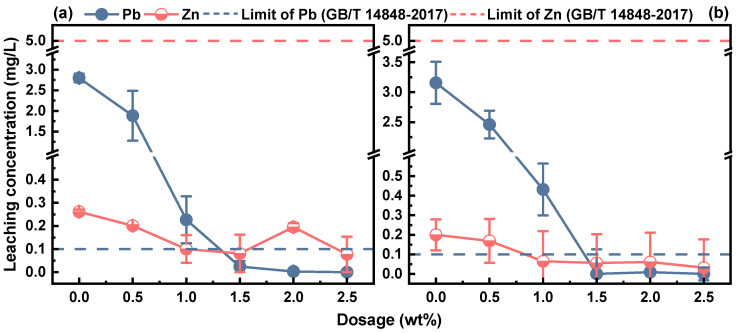
Leaching behavior of Pb and Zn in LDH–FA: (**a**) deionized water as the extractant; (**b**) sulfuric–nitric acid as the extractant.

The nanoscale dispersion of Pb and Zn observed within the LDH-FA structure necessitates a systematic evaluation of HM leaching behavior under different leaching conditions to assess its environmental compatibility for practical applications such as groundwater remediation and soil amendment [36,37]. The leaching concentrations of LDH-FA in deionized water are shown in Figure 4a. Despite the relatively high Pb content in the raw FA, the addition of SDD significantly reduced Pb leaching from LDH-FA. When the dosage reached 1.0 wt%, the Pb concentration fell below the regulatory limit. When the SDD dosage exceeded 1.0 wt%, the Pb concentration further decreased to below 0.05 mg/L. The addition of SDD also reduced the leaching toxicity of Zn from approximately 0.25 mg/L to nearly zero. Zn consistently maintained low leaching levels across all dosages, remaining below regulatory thresholds. This result is notable because Zn was the dominant HM in the raw FA.

The leaching concentrations of Pb and Zn in LDH-FA using a sulfuric–nitric acid solution (simulating acid rain) as the extractant are shown in Figure 4b. The addition of SDD can effectively reduce the leaching risk of HMs from LDH–FA. When the SDD dosage reached 1.5 wt%, the Pb concentration fell below the regulatory limit. With increasing SDD dosage (0–2.5 wt%), the Pb leaching concentration decreased significantly from approximately 3.0 mg/L to below 0.25 mg/L, indicating excellent immobilization performance. Zn maintained consistently low leaching levels across all dosages, despite its high initial content in the raw FA, which may be attributed to its low leachability in the sulfuric-nitric acid environment [38]. As a quantitative benchmark, SDD post-treatment of raw FA under comparable dosage conditions led to Pb leaching concentrations of approximately 0.5 mg/L in deionized water [18] and 0.2–0.8 mg/L in acid rain leachates [30]. Meanwhile, Ca-based LDHs derived from pure reagents were typically assessed for external contaminant removal rather than intrinsic metal stabilization [22]. In the present in situ chelation-crystallization system, Pb concentrations remained below 0.1 mg/L under both leaching scenarios, which confirms the distinctive advantage of the integrated strategy over conventional approaches. In summary, when the SDD dosage is ≥1.5 wt%, the leaching concentrations of Pb and Zn in LDH-FA under both normal rainwater and acid rain conditions meet the Class IV limits of the groundwater quality standard (e.g., for landscape water use), confirming the environmental feasibility of applying LDH-FA in in situ groundwater remediation.

### 3.3. Heavy Metal Speciation and Environmental Risk Assessment

**Figure 5:**
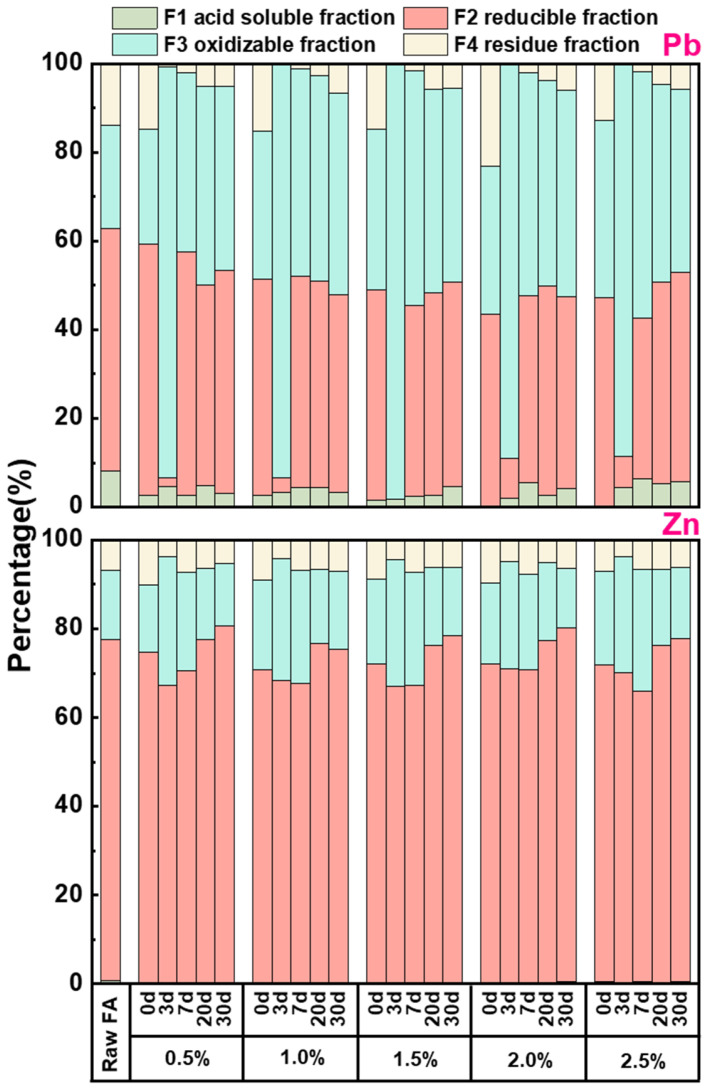
Changes in heavy metal speciation.

Figure 5 illustrates the changes in speciation distribution of Pb and Zn under various SDD dosages (0.5–2.5 wt%) and different aging periods (0, 3, 7, 20, and 30 days). In both Raw FA and treatment groups, HMs are categorized into four fractions: F1 acid-soluble fraction, F2 reducible fraction, F3 oxidizable fraction, and F4 residual fraction.

For Pb, raw FA predominantly contains F1 and F2, indicating high environmental risk. With increasing SDD dosage, the proportion of F1 significantly decreases while F3 and F4 notably increase, particularly at 1.5–2.5 wt% dosages where F3 becomes dominant (approximately 40–50%). The decrease in the proportion of F1 reduced the leaching toxicity of Pb, which is consistent with the leaching test results. With increasing storage time, the proportion of F1 showed a slight increase, but it remained significantly lower than that of the raw FA. Meanwhile, the proportions of F3 and F4 were consistently higher than those in the raw FA. When the SDD dosage was higher than 2.0 wt%, the F2 still maintained a considerable proportion (approximately 40–50%) after 30 days, indicating that Pb stabilization is an ongoing process.

For Zn, the speciation distribution differs from Pb. In raw FA, Zn primarily exists as F2 and F3, with a relatively low F1 acid-soluble fraction, which also explains why Zn has a high total content in raw FA but relatively low leaching. After the formation of LDH-FA, as the SDD dosage increases, F1 further decreases while F4 increases. The impact of aging time on Zn speciation is more complex, but the general trend shows increasing proportions of F3 and F4, indicating a gradual transformation toward more stable forms. Moreover, within 30 days after the addition of the SDD, F1 is almost absent, and the proportions of F3 and F4 remain consistently higher than those in raw FA.

Overall, this study demonstrates that SDD effectively promotes HM transformation from easily leachable forms (F1) to more stable forms (F3 and F4), with this transformation effect enhancing with increased dosage. SDD dosages of 1.5–2.5 wt% exhibit superior HM stabilization effects, not only rapidly reducing F1 proportion but also promoting the formation of long-term stable fractions, thereby effectively reducing the environmental risk of HMs.

**Figure 6:**
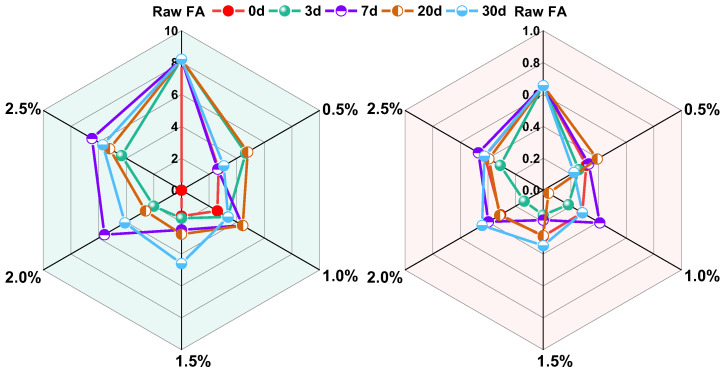
Independent environmental risk assessment of heavy metals.

Figure 6 illustrates the independent environmental risk assessment of Pb and Zn based on the risk assessment code (RAC) method, which evaluates environmental risk levels through the proportion of easily leachable fraction (F1) relative to total HM content.

For Pb, the raw FA contains approximately 8.2% in F1, classifying it as “low risk” according to RAC standards. As the SDD dosage increases, the F1 proportion of Pb decreases significantly. When the SDD dosage is 0.5–1.5 wt%, the RAC of Pb in LDH-FA decreases but remains greater than 1. When the dosage reaches 2.0 wt%, the RAC value drops to 0, reducing the risk level to safe. With increasing aging time, the RAC values of Pb in LDH–FA at each dosage show slight increases, but all remain lower than those of raw FA and within a low-risk range.

For Zn, the RAC value of raw FA is 0.66, categorizing it as “safety.” Although its RAC value is already low and within a safe range, the addition of SDD further reduces the risk level. Overall, within 30 days, the RAC values of Zn in LDH-FA after SDD addition remain below 0.5, indicating that Zn poses no environmental threat. Compared to Pb, Zn shows no variation in risk level across time points, indicating that SDD’s stabilization effect on Zn is less time-dependent than for Pb.

In summary, in practical applications, SDD dosages of 2.0–2.5 wt% represent optimal choices for reducing the environmental risk of HMs in FA. The above results indicate that LDH–FA may present a low environmental risk of Pb after long-term storage, while Zn consistently remains within a safe range.

## 4. Conclusions

This study demonstrated the successful upcycling of hazardous FA into well-crystallized CaFe-LDH nanomaterials featuring a distinctive two-dimensional layered nanostructure, thereby establishing a viable technological pathway for FA resource valorization. The incorporation of SDD during hydrothermal synthesis enabled synergistic immobilization of Pb and Zn within the LDH nanostructure, effectively transforming these HMs from environmentally labile acid-soluble fractions into stable residual phases. At an optimal SDD dosage of 1.5 wt%, the leaching concentration of Pb in both deionized water and simulated acid rain was consistently below 0.1 mg/L, meeting the Class IV limits of the groundwater quality standard and supporting the environmental feasibility of LDH-FA for in situ groundwater remediation scenarios. Risk assessment code (RAC) analysis further revealed that the SDD-modified LDH-FA nanomaterials pose negligible environmental risk over a 30-day assessment period. Collectively, these findings demonstrate that SDD-assisted LDH-FA material represents a rational and scalable strategy for converting hazardous FA into an environmentally compatible functional nanomaterial, offering significant potential for HM pollution control and site remediation.

## Figures and Tables

**Table 1:** The elemental composition of the raw FA.

Oxide	CaO	Cl	Na_2_O	SO_3_	K_2_O	SiO_2_	ZnO	MgO	Fe_2_O_3_	Al_2_O_3_
Content, wt%	53.28	19.88	8.56	8.04	5.52	1.89	0.58	0.56	0.45	0.38

## Data Availability

The original contributions presented in this study are included in the article/Supplementary Materials. Further inquiries can be directed to the corresponding author.

## Supplementary Materials

The following supporting information can be downloaded at https://www.mdpi.com/article/10.3390/nano16110697/s1, Figure S1: Mineral phases in raw fly ash; Figure S2: Mineral phases of LDH materials synthesized with different ratios (FA: FeCl_3_ = 1:1, 2:1, and 3:1); Figure S3: Raw FA and LDH-FA (FA: FeCl_3_ = 1:1, 2:1, and 3:1) Material FTIR properties; Figure S4: Raman spectral characteristics of Raw FA and LDH-FA (FA: FeCl_3_ = 1:1, 2:1 and 3:1) materials; Table S1: The steps of BCR extraction processes; Table S2: Classification of Heavy Metal Environmental Risk Levels by RAC Method; Table S3: The content of heavy metals in the raw FA; Table S4: BET specific surface area, pore volume, and average pore diameter of LDH-FA materials (FA: FeCl_3_ = 3:1) with different SDD dosages.
