# Supplementary material for: Upcycling Municipal Solid Incineration Fly Ash into Layered Double Hydroxide Nanomaterials: Heavy Metal Immobilization and Environmental Risk Assessment
Source: Nanomaterials (Basel). 2026 Jun 3;16(11):697. doi: 10.3390/nano16110697 (PMC13258753; doi:10.3390/nano16110697)
Supplement: Supplementary file 1 [file nanomaterials-16-00697-s001.zip › nanomaterials-4304153-supplementary.pdf]

# Upcycling municipal solid incineration fly ash into Layered Double Hydroxide Nanomaterials: Heavy Metal Immobilization and Environmental Risk Assessment

Yue Zhao [1], Xiaona Wang [2,3], Ze Zhang [4], and Menglan Xu [5,*]

1) State Key Laboratory of Water Resource Protection and Utilization in Coal Mining, China Energy Investment Group, Beijing 102209, China.
2) School of Energy and Environmental Engineering, University of Science and Technology Beijing, Beijing 100083, China.
3) Shunde Innovation School, University of Science and Technology Beijing, Foshan 528399, China.
4) Graduate school of Frontier Sciences, The University of Tokyo, Kashiwa 277-8563, Japan.
5) State Key Laboratory of Iron and Steel Industry Environmental Protection, Central Research Institute of Building and Construction Co., Ltd., MCC Group, Beijing 100088, China.

*Corresponding author: 15376594909@163.com

Table S1. The steps of BCR extraction processes

| Step | Speciation | Extraction solution | Procedure |
|---|---|---|---|
| 1 | Acid soluble fraction(F1) | acetic acid solution [c(HAc) = 0.11mol/L] | 40 ml acetic acid solution, centrifuge after agitating continuously for 16h at room temperature. |
| 2 | Reducible fraction (F2) | hydroxylamine hydrochloride solution[c($NH_2OH \cdot HCl$) = 0.50mol/L] | 40 ml hydroxylamine hydrochloride solution centrifuge after agitating continuously for 16h at room temperature. |
| 3 | Oxidizable fraction (F3) | hydrogen peroxide [30% $H_2O_2$], ammonium acetate solution [c($NH_4Ac$)=1.000mol/L] | 10 ml 30% $H_2O_2$ (adjust PH = 2.0 with $HNO_3$), digested at room temperature for 1 h and then in a water bath at 85 °C for 1h;<br>10 ml of 30% $H_2O_2$ was added repeatedly, heated until 1 ml of the solution remained;<br>40 ml 1 mol/L $NH_4OAc$, centrifuge after agitating continuously for 16h at room temperature. |
| 4 | Residual fraction(F4) | $HNO_3$ (AR), HCl (AR), HF (AR), and hydrogen peroxide [30% $H_2O_2$] | 9ml concentrated nitric acid, 2ml concentrated hydrochloric acid, 3ml hydrofluoric acid, and 1ml hydrogen peroxide, digest the remaining residue |

Table S2. Classification of Heavy Metal Environmental Risk Levels by RAC Method

| RAC index | Environmental risk level |
|---|---|
| RAC≤1 | Safety |
| 1＜RAC≤10 | Low risk |
| 10＜RAC≤30 | Medium risk |
| 30＜RAC≤50 | High risk |
| RAC＞50 | Extremely high risk |

Table S3. The content of heavy metals in the raw FA.

| **HM** | **Ni** | **Cr** | **Cd** | **As** | **Cu** | **Pb** | **Zn** |
|---|---|---|---|---|---|---|---|
| Content, mg/kg | 13 | 62 | 150 | 282 | 286 | 792 | 3936 |

TableS4. BET specific surface area, pore volume, and average pore diameter of LDH-FA materials ($FA: FeCl_3 = 3:1$) with different SDD dosages.

| Sample | Specific surface area （$cm^3/g$） | Pore volume（$cm^3/g$） | Average aperture （nm） |
|---|---|---|---|
| 3:1 (0 wt% SDD) | 18.66 | 0.141 | 30.20 |
| 3:1-0.5 | 17.49 | 0.098 | 22.45 |
| 3:1-1.0 | 20.28 | 0.168 | 33.16 |
| 3:1-1.5 | 18.98 | 0.158 | 33.38 |
| 3:1-2.0 | 17.92 | 0.115 | 25.71 |

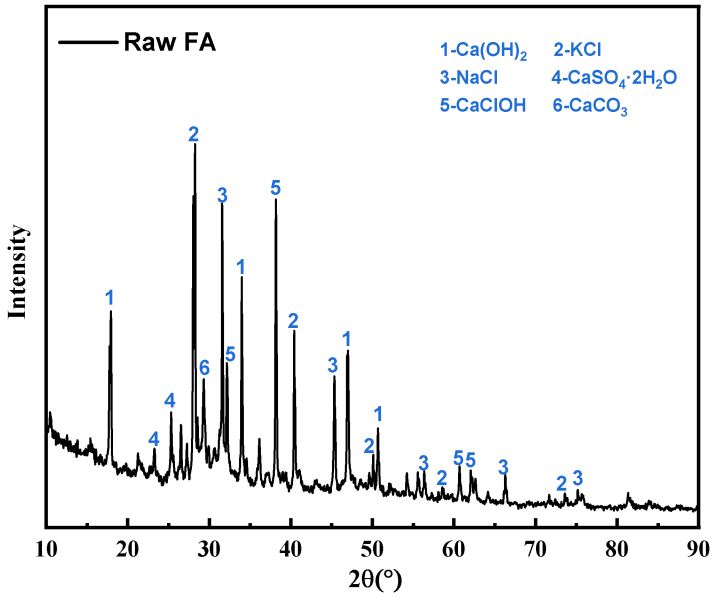


Figure S1. Mineral phases in raw fly ash

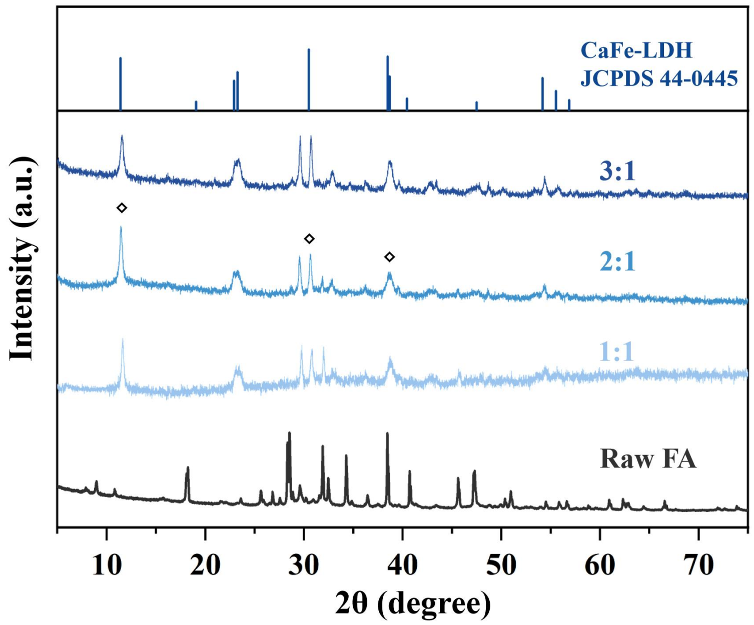


Figure S2. Mineral phases of LDH materials synthesized with different ratios (FA: $FeCl_3$ = 1:1, 2:1, and 3:1)

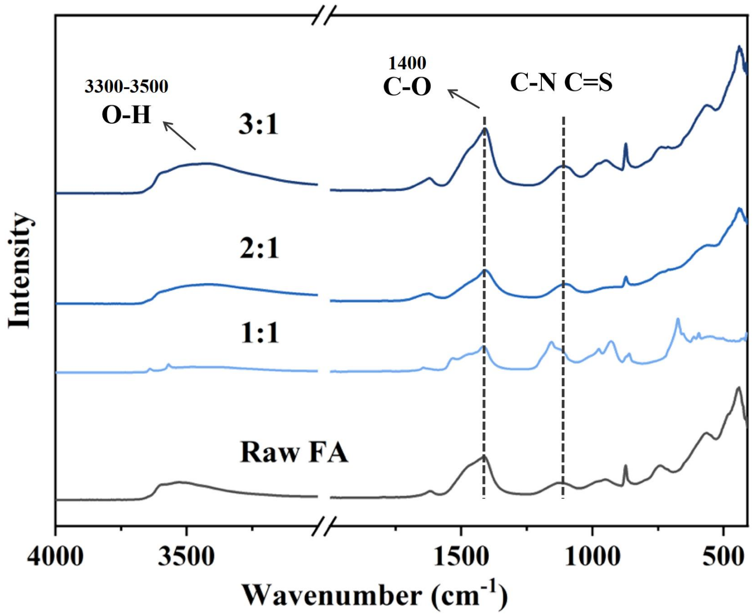


Figure S3. Raw FA and LDH-FA (FA: $FeCl_3$ = 1:1, 2:1, and 3:1) Material FTIR properties

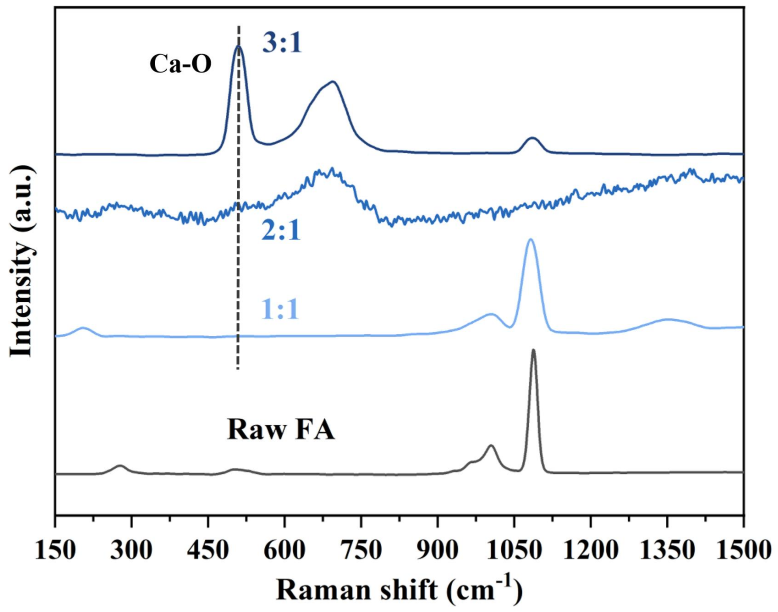


Figure S4. Raman spectral characteristics of Raw FA and LDH-FA (FA: $FeCl_3$ = 1:1, 2:1 and 3:1) materials
